# The influence of obesity, diabetes mellitus and smoking on fuchs endothelial corneal dystrophy (FECD)

**DOI:** 10.1038/s41598-024-61948-w

**Published:** 2024-05-21

**Authors:** S. B. Zwingelberg, B. Lautwein, T. Baar, M. Heinzel-Gutenbrunner, M. von Brandenstein, S. Nobacht, M. Matthaei, B. O. Bachmann

**Affiliations:** 1 Department of Ophthalmology, Faculty of Medicine and University Hospital of Cologne, University of Cologne, Kerpener Street 62, 50937 Cologne, Germany; 2 Faculty of Medicine and University Hospital of Cologne, University of Cologne, Cologne, Germany; 3 Institute for Medical Statistics and Bioinformatics of the University of Cologne, Bachemer Straße 86, 50931 Cologne, Germany; 4 Philipps—University Marburg, Biegenstraße 10, 35037 Marburg, Germany; 5 Department of Urology, Faculty of Medicine and University Hospital of Cologne, University of Cologne, Cologne, Germany; 6 Department of Ophthalmology, Radboud-Universität Nijmegen, Nijmegen, The Netherlands

**Keywords:** Female sex, Genetics and epigenetics, Diabetes mellitus, Obesity, Nutrition, Corneal diseases, Outcomes research, Nutrition, Diabetes, Metabolic syndrome, Obesity, Risk factors

## Abstract

To detect environmental factors, which may be possible risk factors in the disease course of Fuchs’ endothelial corneal dystrophy (FECD). Evaluation of patients with FECD registered in the FECD genetics database of the Center for Ophthalmology, University Hospital Cologne. For the evaluation, disease onset, central corneal thickness, best spectacle corrected visual acuity (BSCVA, logMAR), and modified Krachmer grading (grades 1–6) were correlated with the presence of diabetes mellitus (DM), body mass index (BMI), and smoking behavior. To put the age-related increase in Krachmer grading into perspective, a correction of grading were formed. Depending on the variables studied, differences between groups were examined by Mann–Whitney U test and chi-square test. The significance level was 5%. 403 patients with FECD were included in the analysis. The mean age of the patients was 70.0 ± 10.32 (range 28–96) years. The mean age at diagnosis of those patients was 63.1 ± 13.2 years. The female-to-male ratio was 1.46:1. Patients with a BMI > 30.0 kg/m^2^ developed FECD significantly earlier than patients with a BMI < 30 kg/m^2^, *p* = 0.001. Patients with DM showed significantly more often an Krachmer grade of 5, *p* = 0.015. Smoking had a negative effect on Krachmer grading (*p* = 0.024). Using the mediation analysis, the presence of DM correlated Krachmer Grade 5 (*p* = 0.015), and the presence of DM correlated with BMI > 30.0 kg/m^2^ (*p* = 0.012). In addition to smoking and DM our study shows for the first time that obesity may have a negative impact on the development of FECD. Whether dietary interventions and hormones can influence the development or progression of the disease needs to be investigated in future studies.

Fuchs’ endothelial corneal dystrophy (FECD) is an age related disease leading to the formation of guttae and to endothelial decompensation^1,2^. Patients are typically asymptomatic at early stages when first morphological alterations, such as polymegethism, a variation in the cell size, and pleomorphism, a variation in cell shape. Can be recognized.

FECD is a complex and heterogeneous genetic disease, in which the interaction between genetic and exogenous factors leads to oxidative stress, auto(mito)phagy, unfolded protein response, mitochondrial dysfunction and cellular apoptosis^3^.

There exist two types of Fuchs endothelial corneal dystrophy (FECD): the early onset FECD, which appears in the first decade of life, in contrast to the late onset FECD, which occurs after the 40th decade and shows a higher incidence as the early onset form. The early-onset form and the late-onset form of the FECD are associated with changes in certain genes: the early-onset form is almost associated with changes in the *COL8A* gene on chromosome 1p34.3–32.3. The genetics of the late subtype of the FECD seems to be more complex and heterogeneous: usually there exists a mutation of transcription factor 4 (TCF4) on chromosome 18 with an increase of a CTG triplet repeat. In addition, the transcription factor 8 (TCF8), the ATP/GTP binding protein like 1 (AGBL1), the lipooxygenase homology domain 1 (LOXHD1) as well as the SCL transporter SCL4A11 and the transforming growth factor-beta in the induced form (TGFBI), are affected^4,5^. Epigenetic factors are likewise discussed in both types of the FECD as an additional pathogenetic mechanism^6,7^. FECD is characterized by a progressive decrease in the endothelial cell count (ECC), alterations in the shape and size of the residual cells and formation of corneal guttae. The corneal endothelium located at the basement Descemet membrane is the innermost corneal layer and is important for the clarity of the cornea. Responsible for the pump function and in consequence for the clarity of the cornea is the Na^+^/K^+^ ATPase^8^. Unlike the epithelium, the endothelium does not have self-renewing ability and cannot proliferate. Therefore, cell damage caused by different pathologies, stimulates the remaining endothelial cells to enlarge and migrate to cover the defects, thereby maintaining corneal transparency^4^. Corneal endothelial decompensation leads to „overhydratation” of the cornea, known as corneal edema. The patient may be asymptomatic in the early stages. The progress of corneal edema leads to glare or blurred vision and discomfort or even serve pain caused by folds in the Descemet´s membrane and increase stromal thickness and in the further course bullae^9^.

Although there exist a few studies that have investigated the influence of individual environmental factors that can also accelerate the symptoms and progression of Fuchs' endothelial corneal dystrophy (FECD), no study has yet described and analyzed the interaction of these environmental factors and which one may have the greatest impact. Therefore, our study analyzed possible risk factors, such as gender, smoking, diabetes mellitus (DM) and the body mass index (BMI) in correlation to the Krachmer Grading, which may also have an interactive influence on the epigenetic changes of FECD.

This present study may help to better understand the pathomechanism of FECD also may detect novel therapeutic possible target points for the treatment of FECD in future.

## Methods and collection of clinical data

### Inclusion and exclusion criteria

For this non-randomized single-center analysis 403 patients between the age of 28 and 96 years with a diagnosis of FECD were reviewed from the prospective Fuchs genetic Database of the University of Cologne approved by the Ethics Commission of Cologne University’s Faculty of Medicine (application No. 16-424).

Exclusion criteria were insufficient data and extracorneal visual acuity-limiting conditions including age-related macular degeneration, diabetic retinopathy, macular edema, advanced stages of glaucoma, and amblyopia. Previous eye surgery was also an exclusion criterion: patients who had undergone previous cataract surgery, vitrectomy, pressure-reducing glaucoma surgery, and previous corneal surgery were actively excluded from the study.

### Clinical Information and outcome parameters

For our database we used REDCap, a program for clinical and translational research (Research Electronic Data Capture, Vanderbilt University, Nashville, 2004): Demographic and clinical data including age at diagnosis, gender, as well as the best spectacle-corrected visual acuity (BSCVA, logMAR), the central corneal thickness (CCT) (by using Oculus, Pentacam), the Krachmer grading based on slit-lamp examination^10^, the presence of DM and the smoking behavior as well as the body height and weight, were analyzed.

The smoking behavior was described in pack years. The body mass index (BMI) was calculated from the patients’ height and weight with the formula BMI = m l^2^ (kg/m^2^). The BMI was classified according to the WHO guideline (1: BMI < 18.5 kg/m^2^, underweight; 2: BMI 18.5—24.9 kg/m^2^, normal weight; 3: BMI 25.0—29.9 kg/m^2^, overweight; 4: BMI ≥ 30.0 kg/m^2^, obesity^11^.

### Statistical analyses

The collected data, including the descriptive data, was analyzed by SPSS version 25.0 software (SPSS, Chicago, Illinois) for Windows.

The patient cohort was evaluated by using descriptive statistics. The mean values of the metric variables were shown ± one standard deviation and the absolute frequencies with the percentages in brackets.

The analysis included the following variables: Age of patients, age of diagnosis, gender, presence of diabetes mellitus, smoking behavior, body mass index (BMI), Krachmer grading, central corneal thickness (CCT, measured with Pentacam, Oculus, Wetzlar, Germany) and best spectacle corrected visual acuity (BSCVA). The variable BSCVA was converted into the unit logMAR prior to statistical analysis. The significance level was 5%. The variables were analyzed by using Kolmogorov–Smirnov test and Shapiro–Wilk test for normal distribution.

In order to be able to use the correct statistical test procedures, we first analyzed the distribution pattern of the individual parameters.

A Spearman correlation was used to investigate whether there was a relationship between Krachmer grading (transposed to grades 1–6) and age. To put the age-related increase in Krachmer grading into perspective, two approaches to age correction of grading were performed: Method 1—by quotient formation (grading by age); Method 2—by ordinal regression. The age corrected/modified Krachmer grading was not normally distributed, so in the following procedure it was always examined by using non-parametric methods. A Mann–Whitney U test was used to investigate whether gender makes a difference in the age of diagnosis. Pearson's chi-square test was applied to analyze whether there are differences in grading between the genders. A one-way analysis of variance (ANOVA) was applied to test, if there was a correlation between the grading and the CCT or BSCVA.

The risk factor “smoking” could be expressed by two influencing variables: first, as a dichotomous (nominal) variable with the groups "smokers and former smokers" and "non-smokers". Secondly, pack-years were presented as a metric variable, calculated as the product of the number of packs smoked per day and the number of years smoked. Smokers and former smokers with ≥ 70 Packyears were examined for a difference regarding the modified Krachmer grading by using chi-square test or Fisher's exact test compared to non-smokers, and with respect to the age of diagnosis using a Mann–Whitney U test. The Packyears were examined for a correlation with the grading by applying ANOVA.

The “body mass index” (BMI) as a risk factor is determined by body weight and height in kg/m^2^. A BMI of 30 or more indicates that the patient is obese. The BMI was also defined by two influencing variables. Firstly, as a dichotomous (nominal) variable with the groups "BMI < 30" and "BMI ≥ 30". Secondly, as a metric variable with the BMI value itself. The BMI value was examined for a correlation with the modified Krachmer grading by applying ANOVA. The variables BMI and diabetes mellitus were analyzed for a correlation by using a cross-tabulation and chi-square test. The groups of the diabetes mellitus variable were examined for differences in BMI by evaluating with Mann–Whitney U test.

The risk factor “diabetes mellitus” was represented by a dichotomous (nominal) influence variable with the groups "diabetes mellitus" and "no diabetes mellitus".

The comparison of the groups within the individual risk factors were analyzed in respect to the age at diagnosis, CCT and BSCVA by using Mann–Whitney U test, and for the modified Krachmer grading by using chi-square test and Fisher's exact test, respectively. The Packyears were examined for a correlation with the grading by applying ANOVA.

### Ethical approval

All procedures performed in studies involving human participants were in accordance with the ethical standards of the institutional research committee and with the 1964 Helsinki declaration and its later amendments or comparable ethical standards.

### Informed consent

For this type of study, formal consent is by the Ethics Commission of Cologne University’s Faculty of Medicine (application No. 16-424). All study participants and/or their legal guardians have given their informed consent to this study.

## Results

### Demographics

The patient collective consisted of n = 403 FECD patients with a female-to-male ratio of 1.46:1 (239 (59.3%):164 (40.7%)). The mean age of patients at examination was 70.0 ± 10.32 (range 28–96) years. The median age at diagnosis was 63.1 ± 13.2 years. The female FECD patients was affected on average with 62.8 versus 63.7 years in the FECD male patients, without a significance of *p* = 0.502. Lower age at diagnosis of FECD correlated with higher modified Krachmer grading, *p* = 0.005. An overview of the patient collective can be found in Tables 1 and 2.

**Table 1:** Presentation of the patient collective I.

	N	Percentage share (%)
Non-smoker	205	50.9
Smokers and former smokers	198	49.1
Patients without diabetes mellitus	350	86.8
Patients with diabetes mellitus	53	13.2
Patients with a BMI < 30	316	80.8
Patients with a BMI ≥ 30	75	19.2

**Table 2:** Presentation of the patient collective II.

	Age	Age at diagnosis	Grading	CCT (µm)	Visual acuity (logMAR)	Packyears	BMI
N	403	402	396	387	175	198	391
Mean value	70.00	63.13	5.62	641.50	0.45	20.52	26.67
Std. deviation	10.19	13.20	0.69	92.79	0.29	21.80	4.55
Median	71.00	65.00	6.00	629.00	0.40	15.00	25.95
Minimum	28.00	2.00	1.00	438.00	0.00	0.20	17.92
Maximum	96.00	94.00	6.00	1225.00	1.30	125.00	50.17

### Clinical data

#### Smoking versus non-smoking

198 (49.1%) study patients were smokers or former smokers (group I) and 205 (50.9%) study patients were non-smokers (group II). On average, 20.52 ± 21.80 pack years were smoked in group I. The mean age at diagnosis in group I was 61.96 ± 13.51 years and 64.27 ± 12.82 years in group II, *p* = 0.063. The proportion of smokers in men was significantly higher than in women, *p* = 0.004. Smoking had a negative effect on Krachmer grading after age correction by method 2, when comparing smokers/former smokers (5.58 ± 0.69) versus non-smokers (5.65 ± 0.61), *p* = 0.024. The number of packyears was statistically not related to modified Krachmer grading with *p* = 0.514.

The group of smokers and former smokers had an average BSCVA of 0.47 ± 0.29 logMar and the median was 0.40. The group of non-smokers had an average BSCVA of 0.44 ± 0.30 logMar with a median of 0.40. The difference between the groups was statistically insignificant, *p* = 0.247 (compare Table 3).

**Table 3:** Results risk factor smoking.

		Age	Age at diagnosis	Grading	CCT (µm)	Visual acuity (logMAR)
Smokers and former smokers	N	198	198	195	191	94
	Mean value	69.38	61.96	5.58	629.7	0.47
	Median	70.00	64.00	6.00	619.0	0.40
	Std. deviation	9.67	13.51	0.74	75.73	0.29
	Minimum	39.00	2.00	1.00	438.0	0.00
	Maximum	96.00	94.00	6.00	1161.0	1.30
Non-smoker	N	205	204	201	196	81
	Mean value	70.61	64.27	5.65	652.9	0.44
	Median	73.00	66.00	6.00	637.5	0.40
	Std. deviation	10.66	12.82	0.63	105.76	0.33
	Minimum	28.00	5.00	4.00	501.0	0.00
	Maximum	90.00	86.00	6.00	1225.0	1.30
	Significance		*p* = 0.063	*p* = 0.373	*p* = 0.024	*p* = 0.247

### Body mass index (BMI)

The distribution of BMI in our cohort was 38.2% (n = 154) with normal weight (BMI ≤ 18.5—24.9 kg/m^2^), 40% (n = 161) with overweight (BMI of 25.0—29.9 kg/m^2^) and 21.8% (n = 88) had obesity (BMI > 30.0 kg/m^2^).There was a significant difference in the mean age at diagnosis of FECD: The average age of diagnosis in the group of patients with a BMI < 30 was 64.40 ± 12.31 years with a median of 66 years. In the group of patients with a BMI ≥ 30, the average was 58.59 ± 15.06 years and the median was 60 years. This difference was statistically significant with *p* = 0.001 (compare Table 4). The difference between patients with a BMI > 30 kg/m^2^ and patients with a BMI < 30 kg/m^2^ with regard to their Krachmer grading (without age-correction) showed statistically no significance (BMI < 30: 5.65 ± 0.69 versus BMI ≥ 30: 5.54 ± 0.62, *p* = 0.053) and was also not significantly different even after age correction using quotient formation and ordinal regression.

**Table 4:** Results risk factor BMI.

		Age	Age at diagnosis	Grading	CCT (µm)	Visual acuity (logMAR)
BMI < 30	N	316	315	310	303	136
	Mean value	71.02	64.40	5.65	641.5	0.46
	Median	73.00	66.00	6.00	627.0	0.40
	Std. deviation	9.86	12.31	0.69	95.32	0.30
	Minimum	28.00	5.00	1.00	438.0	0.00
	Maximum	96.00	94.00	6,00	1225.0	1.30
BMI ≥ 30	N	75	75	74	73	33
	Mean value	66.33	58.59	5.54	635.2	0.46
	Median	67.00	60.00	6.00	638.0	0.40
	Std. deviation	10.29	15.06	0.62	62.85	0.26
	Minimum	39.00	2.00	4.00	501.0	0.10
	Maximum	92.00	84.00	6.00	804.0	1.30
	Significance		*p* = 0.001	*p* = 0.053	*p* = 0.760	*p* = 0.643

The mean CCT in patients with a BMI < 30 was 641.45 ± 95.32 µm with a median of 627 µm. The CCT in patients with a BMI ≥ 30 was 635.15 ± 62.85 µm and the median was 638 µm. This difference was not statistically significant, *p* = 0.760. BSCVA in the patients with BMI < 30 was 0.46 ± 0.30 logMar and its median 0.40 logMar. In the patients with a BMI ≥ 30, the mean BSCVA was 0.46 ± 0.26 logMar and the median was 0.40 logMar. The difference between both groups was statistically insignificant, *p* = 0.643.

### Diabetes mellitus (DM) versus non diabetes mellitus (Non-DM)

13.2% (n = 53) study patients had DM. Of these, n = 49 (92.5%) were type 2 diabetics and n = 3 (5.7%) type 1. Type of DM of one patient could not be confirmed. DM had no effect on the age at diagnosis (64.59 ± 13.76 years with DM versus 62.91 ± 13.12 without DM, *p* = 0.193). The diabetic group had a mean CCT of 644.69 ± 117.44 µm and the median was 613 µm. The non-diabetic group had a CCT of 641.02 ± 88.65 µm with a median of 630 µm (compare Table 5). This difference was not statistically significant, *p* = 0.440. The BSCVA in the diabetic group was 0.49 ± 0.31 and the median was 0.40. The non-diabetic group had a mean BSCVA of 0.45 ± 0.29 and with median of 0.40. This difference was statistically insignificant with *p* = 0.503. The mean value in modified Krachmer grading by using method 1 in the diabetics was 5.63 ± 0.60 and the median was grade 6, while in the non-diabetics the mean value was 5.61 ± 0.70 and the median was grade 6, *p* = 0.015.

**Table 5:** Results risk factor Diabetes mellitus (DM).

		Age	Age at diagnosis	Grading	CCT (µm)	Visual acuity (logMAR)	BMI
DM: Yes	N	53	53	49	51	27	51
	Mean value	72.34	64.59	5.63	644.7	0.49	28.76
	Median	75.00	66.00	6.00	613.0	0.40	28.37
	Std. deviation	8.97	13.76	0.60	117.44	0.31	4.61
	Minimum	47.00	8.00	3.00	477.0	0.10	20.45
	Maximum	90.00	82.00	6.00	1158.0	1.30	41.32
DM: No	N	350	349	347	336	148	340
	Mean value	69.65	62.91	5.61	641.0	0.45	26.36
	Median	71.00	65.00	6.00	630.5	0.40	25.62
	Std. deviation	10.33	13.12	0.70	88.65	0.29	4.47
	Minimum	28.00	2.00	1.00	438.0	0.00	17.92
	Maximum	96.00	94.00	6.00	1225.0	1.30	50.17
	Significance		*p* = 0.193	*p* = 0.015	*p* = 0.440	*p* = 0.503	*p* < 0.001

### Correlation of diabetes mellitus and body mass index

The comorbidities diabetes mellitus and obesity were statistically significantly related, *p* = 0.006. The average BMI in the diabetic group was 28.76 ± 4.61 and the median was 28.37. In the non-diabetic group, the average BMI was 26.36 ± 4.47 with a median of 25.62. This difference was statistically significant, *p* < 0.001.

### The influence of age at diagnosis on Krachmer grading

According to our analyses a lower age at diagnosis correlated significantly with a higher modified Krachmer grading by using method 2 (Rho = 0,095, *p* < 0.005).

## Discussion

Our study allows four important conclusions to be drawn:

(i) The environmental factor smoking has a negative impact on the modified Krachmer grade.
(ii) The age of onset of FECD significantly correlates with the degree of obesity.
(iii) The comorbidity diabetes mellitus has a significant impact on the modified Krachmer grade.
(iv) FECD has a greater incidence in women, which are predominantly affected postmenopausal.

Smoking showed a negative effect on the development of FECD. Smoking leads to an increase in free radicals and a decrease in antioxidants in the blood and subsequently in ocular tissues^12–19^. As a result, smokers' eyes are at increased risk for free radical damage and oxidative stress, leading to increased apoptosis of endothelial cells, associated with increased corneal guttae and a higher age corrected Krachmer grading^12–19^. Our findings are consistent with the results of the "Reykjavik Eye Study", in which it was shown that smokers with 20 pack years have a twofold increased risk for the development of cornea guttata^18^.

We were also able to demonstrate that a BMI ≥ 30 is associated with an earlier onset of FECD. Moreover, a BMI ≥ 30 was correlated with the comorbidity diabetes mellitus. The presence the comorbidity diabetes mellitus was associated with the presence of a high Krachmer grade. Since the vast majority of DM patients in our study suffer from DM type II, the metabolically associated type, we assume that metabolic changes contribute to the onset of FECD. The specific pathophysiology of how DM influences FECD remains unknown^20–27^. Besides a pathological effect on function and morphology of hyperglycemia on corneal endothelial cells the influence of an increased HbA1c glycosylation on corneal collagen fibrils has been discussed^28^. Moreover, an increased intrastromal concentration of sorbitol would increase the corneal thickness by osmotic aqueous retention^29^.

Hyperglycemia has been also reported to be associated with CEC morphological changes^20,21^, increased CEC permeability, and decreased pump rates of Na^+^/K^+^ ATPase^22^. Hyperglycemia has also been shown to increase production of mitochondrial superoxide, leading to DNA damage and depletion of the cellular antioxidant nicotinamide adenine dinucleotide^23,24^. These findings suggest that additional oxidative stress from diabetes may lead to accelerated CEC death in FECD patients, where CEC homeostasis is already impaired^25–27^.

Additionally, fatty tissue leads pathophysiological to an insulin-resistance, especially in patients with DM. The higher the proportion of adipose tissue in patients with a higher BMI, the stronger the insulin resistance and therefore insulin cannot develop any catabolic effects.

Cholesterol levels are pathophysiologically elevated in patients with a higher BMI. It can be assumed that nutrition plays a yet unknown, but important factor in the pathogenesis of FECD.

FECD manifests in the sixth decade of life with a female-male ratio of 1.46:1 according our study, which stands in concordance with other studies, where higher ratios are also described (e.g. 3,5:1 from Afshari et al., 2006)^30–32^. Although the higher prevalence in women is commonly described in other independent studies, the reasons still remain unexplained. Since adipose tissue contains an increased number of aromatic enzymes, which catalyze the synthesis of cholesterol to estrogen in the ten-step steroid synthesis changes in sex hormones could also be a contributing factor to the development of FECD^33^.

Liu and Miyajima et al. showed in their in-vitro study, that estrogen metabolites generate oxidative stress in mitochondria which induces DNA damage and apoptosis in endothelial cells with FECD^34^. They also showed, that those cells have a lower expression of NAD(P)H-dehydrogenase, which serves as an anti-oxidative agent in healthy cells. In summary, in FECD the oxidative-antioxidative balance is challenged by the influence of toxic sex hormone metabolites.

In consideration, that the white fatty tissue in patients with a higher BMI has an independent estrogen production via the physiological enzyme aromatase^33^, the earlier onset of FECD in obese patients might also be explained by the oxidative stress conducted by estrogen-metabolites. In a recent study of Jurkunas et al. 2024 it was demonstrated that the endo-degenerative phenotype is driven by estrogen metabolite-dependent CEC loss that is exacerbated in the absence of NQO1; thus, explaining the mechanism accounting for the higher incidence of FECD in females^35^.

In the light of our clinical observations, further studies on the hormonal status of FECD patients should be conducted in combination with metabolic and nutritional analysis.

## Conclusion

We were able to demonstrate that smoking and DM are risk factors for the development of more severe stages in FECD. Moreover, we could show for the first time that obese individuals develop FECD significantly earlier than non-obese affected individuals. Together with gender as a risk factor, this may indicate a role of glucose metabolism and the influence of estrogens in the development of FECD. Further investigation of these potential risk factors would be of value, as they could be influenced by diet or medication.

## Data availability

The data sets used and/or analyzed in this study are available on request from the corresponding author.
